# Supplementary material for: A Life Cycle Assessment Study of the Impacts of Pig Breeding on the Environmental Sustainability of Pig Production
Source: Animals (Basel). 2024 Aug 22;14(16):2435. doi: 10.3390/ani14162435 (PMC11350755; doi:10.3390/ani14162435)
Supplement: Supplementary file 1 [file animals-14-02435-s001.zip › animals-3128924-supplementary.pdf]

Table S1. Pig production system data and units of measurement for LCA.	
Sow performance	Litter performance
Annual number of sows	Piglets born alive per litter
Annual number of gilts	Farrowing mortality rate (%)
Replacement rate (%)	Lactation mortality rate (%)
Sow mortality rate (%)	Piglets weaned per litter
Number and weight (kg) of culled sows	Pigs weaned per sow per year
Litters per sow per year	Average weight of weaned piglet (kg)
Non-productive days per litter	Average weaning age (days)
Sow barn feed usage	
Gestation and lactation feed intake (kg/year)	Feed composition
Drinking water consumption (l/year)	
Wean-to-finish pig performance	
Average start weight (kg)	Feed intake (kg/day)
Average end weight (kg)	Feed conversion ratio
Growth rate (kg/day)	Finishing mortality rate (%)
Feed composition (overall or by feeding phase)	
Utilities	Manure management
Electricity (fans, lights, etc; kWh)	Type of manure system (assumed deep pit)
Natural gas, propane or other gas (CCF)	Annual volume of manure managed
Chemicals for cleaning	Average nitrogen and phosphorus content
Cooling and cleaning water	Volatile solids content of excreted manure
Veterinary services incl. professional services and pharmaceuticals ($; from CMU I/O tables)	
Research services to estimate the R&D inputs from genetics research ($; from CMU I/O tables)	

Table S2. Diet composition (%) for the sow barn.

	gestation		lactation	
Corn	59.156		44.928	
DDGS	30.000		30.000	
Soybean meal	7.034		17.439	
Soybean oil	-		4.004	
Limestone 36%	1.775		1.677	
Monocal 21.0%	0.912		0.731	
Salt	0.500		0.316	
Vitamin-mineral premix	0.225		0.225	
L-Lysine HCl 78.8%	0.230		0.440	
Choline chloride 60%	0.127		0.127	
Threonine 98.5%	0.041		0.094	
Tryptophan 98.5%	-		0.012	
Methionine 99% DL	-		0.007	
	PIC	ind. avg.	PIC	ind. avg.
Feed intake (kg/sow/year)	673	863	373	338

Table S3. Diet composition (%) for the nursery (N1 to N3) and finisher (F1 to F5) phases.
Entries such as 00 / 00 represent PIC / industry average.

Phase	N1	N2	N3	F1	F2	F3	F4	F5
Start weight (kg)	6.0 / 5.9	6.8	12	25	41	59	82	104
End weight (kg)	6.8	12	25	41	59	82	104	129 / 128
Feed intake (kg/pig)	0.89 / 0.91	5.7 / 6.5	21 / 22	30 / 32	42 / 46	61/ 64	69 / 74	84 / 86
Corn	39.67	43.3	58.64	57.63	55.33	63.45	74.78	82.16
Oat Groats	12.5	10	-	-	-	-	-	-
Corn DDGS 6-9% oil	-	-	5	15	20	20.4	10.02	3.7
Extruded soybean meal	8.23	6.18	-	-	-	-	-	-
Soybean meal 47%	17	20	29.12	24.66	17.03	13.79	12.98	12.03
Fish meal	5	5	-	-	-	-	-	-
L-Lysine HCl 78.8%	0.54	0.49	0.54	0.38	0.40	0.35	0.28	0.25
Methionine 99% DL	0.28	0.26	0.23	0.09	0.02	-	-	-
Threonine 98.5%	0.25	0.23	0.24	0.09	0.06	0.04	0.04	0.04
Tryptophan 98.5%	0.06	0.06	0.06	0.01	0.02	0.02	0.01	0.01
Valine 96.5% L	0.10	0.09	0.12	-	-	-	-	-
Whey dried food grade 72%	13.72	10.98	-	-	-	-	-	-
Soybean oil	0.75	1.30	3.24	-	-	-	-	-
Minerals and vitamins	1.57	1.71	2.78	2.14	2.14	1.95	1.89	1.81
Others	0.317	0.387	0.025	-	-	-	-	-

Supplementary S4. Conversion factors to calculate whole-enterprise FCR.

Abbreviations used in this Appendix are as follows. **ADF**: average daily feed intake (kg/day); **ADG**: average daily gain (kg/day); **DAYS**: number of days on feed; **$DAYS_{POMRT}$**: number of DAYS for animals that die before slaughter; **EWT**: end weight (kg); **FCR**: feed conversion ratio (kg feed/kg live weight gain); **FCR_{we}**: whole-enterprise FCR (kg feed/kg market weight); **FI_{direct}**: cumulative feed intake per market pig (kg); **$FI_{maternal}$**: sow feed intake (kg/market pig); **GHG**: greenhouse gas; **LSY**: number of litters per sow per year; **NSB**: number of stillborn piglets per litter; **POMRT**: post weaning mortality rate; **PRMRT**: pre-weaning mortality rate; **sowFI**: cumulative sow feed intake (kg/sow/year); **TNB**: total number of piglets born per litter.

Consider a fixed-time grower-finisher scenario with a fixed number of DAYS from weaning to slaughter; live end weight including losses is then (simplified somewhat by ignoring variation in weaning weight):

$$\text{EWT} = (1 - \text{PRMRT}) \times (1 - \text{POMRT}) \times \text{ADG} \times \text{DAYS}$$

The direct component of whole-enterprise cumulative feed intake per market pig is the cumulative feed intake of that liveborn grower-finisher pig itself, including losses:

$$\text{FI}_{\text{direct}} = (1 - \text{PRMRT}) \times [\ \text{POMRT} \times \text{DAY}_{\text{POMRT}} + (1 - \text{POMRT}) \times \text{DAYS}\ ] \times \text{ADF}$$

The maternal component of whole-enterprise cumulative feed intake per slaughter pig is:

$\text{FI}_{\text{maternal}} = \frac{\text{sowFI}}{\text{LSY} \times (\text{TNB} - \text{NSB})}$, with sowFI in kg feed per sow per year.

The whole-enterprise FCR per liveborn market pig is then FCR_{we} :

$$\text{FCR}_{\text{we}} = \frac{\text{FI}_{\text{direct}} + \text{FI}_{\text{maternal}}}{\text{EWT}} =$$

$$= \frac{(1 - \text{PRMRT}) \times [\ \text{POMRT} \times \text{DAY}_{\text{POMRT}} + (1 - \text{POMRT}) \times \text{DAYS}\ ] \times \text{ADF} + \frac{\text{sowFI}}{\text{LSY} \times (\text{TNB} - \text{NSB})}}{(1 - \text{PRMRT}) \times (1 - \text{POMRT}) \times \text{DAYS} \times \text{ADG}}$$

The numerator and the denominator represent the whole-enterprise cumulative feed intake per liveborn market pig and the live end weight of that market pig, respectively; both accounting for pre- and postweaning losses.

Differentiation with respect to each trait of interest gives the change in FCR_{we} due to a unit change in that trait (for example, because of genetic improvement) while keeping all the other traits unchanged. We would prefer those changes in FCR_{we} to be negative.

$$\frac{dFCR_{we}}{dADG} = \frac{-FCR_{we}}{ADG}$$

$$\frac{dFCR_{we}}{dADF} = \frac{FI_{direct}}{EWT \times ADF}$$

$$\frac{dFCR_{we}}{dPOMRT} = \frac{(1 - PRMRT) \times DAY_{POMRT} \times ADF + FI_{maternal}}{(1 - POMRT) \times EWT}$$

$$\frac{dFCR_{we}}{dPRMRT} = \frac{FI_{maternal}}{(1 - PRMRT) \times EWT}$$

$$\frac{dFCR_{we}}{dTNB} = \frac{-sowFI}{LSY \times EWT \times (TNB - NSB)^2}$$

$$\frac{dFCR_{we}}{dNSB} = \frac{-dFCR_{we}}{dTNB}$$

A similar approach was described earlier by Amer et al. (2018) and applied by Alfonso (2019).

Example values for the input parameters: ADF = 2.329 kg/d; ADG = (126.7 – 5.75) / DAYS = 0.724 kg/d; DAY_{POMRT} = 167 / 2 = 83.5 d; DAYS = 187 – 20 = 167 d; LSY = 2.39 litters; NSB = 1.40 piglets/litter; POMRT = 0.1283; PRMRT = 0.1644; sowFI = 1000 kg/yr; TNB = 14.43 piglets/litter. From that, EWT = 88.07 kg; FI_{direct} = 304.15 kg; $FI_{maternal}$ = 32.11 kg; FCR_{we} = 3.82 kg/kg. With those values, the derivatives work out as follows:

$$\frac{dFCR_{we}}{dADG} = \frac{-3.82}{0.724} = -5.276 \text{ kg/kg per kg/d}$$

$$\frac{dFCR_{we}}{dADF} = \frac{304.15}{88.07 \times 2.329} = 1.4828 \text{ kg/kg per kg/d}$$

$$\frac{dFCR_{we}}{dPOMRT} = \frac{(1 - 0.1644) \times 83.5 \times 2.329 + 32.11}{(1 - 0.1283) \times 88.07} =$$

$$= 2.5350 \text{ kg/kg per proportion, i.e. } 0.02535 \text{ kg/kg per \% mortality}$$

$$\frac{dFCR_{we}}{dPRMRT} = \frac{32.11}{(1 - 0.1644) \times 88.07} =$$

$$= 0.4363 \text{ kg/kg per proportion, i.e. } 0.004363 \text{ kg/kg per \% mortality}$$

$$\frac{dFCR_{we}}{dTNB} = \frac{-1000}{2.39 \times 88.07 \times (14.43 - 1.40)^2} = -0.0280 \text{ kg/kg per piglet/litter}$$

$$\frac{dFCR_{we}}{dNSB} = -\frac{dFCR_{we}}{dTNB} = 0.0280 \text{ kg/kg per piglet/litter}$$

Supplementary S5. Inventory and pedigree matrix.

Table S5-1. Elements of the inventory for Scenario 1 (baseline comparison of PIC genetics to the North American industry average, both in 2021) with indicator scores (for reliability, completeness, temporal correlation, geographical correlation, and technological correlation) different from [1 1 1 1 1].

Flow	Scores
Ammonia	[2 2 1 2 2]
Calcium chloride	[3 3 3 3 3]
Carbon dioxide - fossil	[2 2 1 2 2]
Carbon monoxide	[2 2 1 2 2]
Dinitrogen monoxide	[2 2 1 2 2]
Ethane-1-1-1-2-tetrafluoro - HFC-134a	[2 2 1 2 2]
Methane	[2 2 1 2 2]
Nitrogen oxides	[2 2 1 2 2]
Occupation - arable	[2 2 1 2 2]
Particulate matter < 2.5 µm	[2 2 1 2 2]
Particulate matter > 10 µm	[2 2 1 2 2]
Research services	[2 2 1 2 2]
Sulfur dioxide	[2 2 1 2 2]
VOC - volatile organic compounds	[2 2 1 2 2]
Water - unspecified natural origin - USA	[2 2 1 2 2]
Zinc monosulfate	[3 3 3 3 3]

Table S5-2. Elements of the inventory for Scenario 2 (forecast of PIC genetics in 2030 compared to 2021) with indicator scores (for reliability, completeness, temporal correlation, geographical correlation, and technological correlation) different from [1 1 1 1 1].

Flow	Scores
Artificial insemination - semen dose	[2 1 1 1 2]
Diesel - burned in agricultural machinery	[2 1 1 1 2]
Electricity - medium voltage	[2 1 1 1 2]
Grow ration - per pig	[2 1 1 1 1]
Liquid manure spreading - by vacuum tanker	[2 1 1 1 2]
Particulate matter < 2.5 µm	[2 1 1 1 2]
Particulate matter < 10 µm	[2 1 1 1 2]
Propane - burned in building machine	[2 1 1 1 2]
Sow ration - piglets per sow per year	[2 1 1 1 1]
Tap water	[2 1 1 1 2]
Transport - tractor and trailer - agricultural	[2 1 1 1 2]
Utilities	[2 1 1 1 2]
Veterinary services	[2 1 1 1 2]

Supplementary S6. Results of the forecast and the baseline comparison.

Table S6-1. Estimated impacts for Scenario 1 (forecast of PIC genetics in 2030 compared to 2021) and Scenario 2 (baseline comparison of PIC genetics to the North American industry average, both in 2021) under the ReCiPe-2016 framework. Impact categories marked with ✓ are among the 14 categories shared by ReCiPe and PEF. Entries in **bold text** are statistically significant (P < 0.05)

**Framework ReCiPe-2016**		PIC 2021 / industry average 2021						PIC 2030 / PIC 2021					
		ReCiPe-E		ReCiPe-H		ReCiPe-I		ReCiPe-E		ReCiPe-H		ReCiPe-I	
Impact category		cutoff	APOS	cutoff	APOS	cutoff	APOS	cutoff	APOS	cutoff	APOS	cutoff	APOS
Fine particulate matter	✓	**0.920**	**0.920**	**0.920**	**0.920**	0.929	0.929	**0.924**	**0.924**	**0.924**	**0.924**	0.920	0.920
Fossil resource scarcity	✓	**0.925**	**0.925**	**0.925**	**0.925**	**0.925**	**0.925**	**0.926**	**0.926**	**0.926**	**0.926**	**0.926**	**0.926**
Freshwater ecotoxicity	✓	0.928	0.928	0.928	**0.929**	0.928	**0.929**	0.934	0.934	0.934	0.934	0.934	0.934
Freshwater eutrophication	✓	**0.927**	**0.927**	**0.927**	**0.927**	**0.927**	**0.927**	**0.932**	**0.932**	**0.932**	**0.932**	**0.932**	**0.932**
Global warming	✓	**0.925**	**0.925**	**0.925**	**0.925**	**0.926**	**0.926**	**0.928**	**0.928**	**0.930**	**0.930**	**0.930**	**0.930**
Carcinogenic toxicity	✓	0.930	0.930	0.929	0.929	**0.925**	**0.925**	0.922	0.922	0.924	0.924	0.927	0.927
Non-carcinogenic toxicity	✓	0.928	0.928	0.927	0.927	0.929	0.929	0.932	0.932	0.934	0.934	0.933	0.933
Ionizing radiation	✓	0.932	0.932	0.934	0.934	**0.935**	0.934	0.916	0.916	0.898	0.898	0.896	0.897
Land use	✓	**0.931**	**0.931**	**0.931**	**0.931**	**0.931**	**0.931**	**0.935**	**0.935**	**0.935**	**0.935**	**0.935**	**0.935**
Marine ecotoxicity		0.928	0.928	0.929	**0.929**	0.928	**0.928**	0.932	0.931	0.934	0.933	0.933	0.933
Marine eutrophication	✓	**0.927**	**0.927**	**0.927**	**0.927**	**0.927**	**0.927**	**0.934**	**0.934**	**0.934**	**0.934**	**0.934**	**0.934**
Mineral resource scarcity	✓	**0.926**	0.926	**0.926**	0.926	**0.926**	0.926	0.929	0.929	0.929	0.929	0.929	0.929
Ozone formation, human health		**0.928**	**0.928**	**0.928**	**0.928**	**0.928**	**0.928**	**0.932**	0.932	**0.932**	**0.932**	**0.932**	**0.932**
Ozone formation, terrestrial ecosystems		**0.928**	**0.928**	**0.928**	**0.928**	**0.928**	**0.928**	**0.932**	0.932	**0.932**	**0.932**	**0.932**	**0.932**
Ozone depletion	✓	**0.925**	**0.925**	**0.925**	**0.925**	**0.925**	**0.925**	**0.934**	**0.934**	**0.934**	**0.934**	**0.934**	**0.934**
Terrestrial acidification	✓	**0.919**	**0.919**	**0.919**	**0.919**	**0.919**	**0.919**	**0.924**	**0.924**	**0.924**	**0.924**	**0.924**	**0.924**
Terrestrial ecotoxicity		**0.928**	0.928	**0.928**	**0.928**	0.928	**0.927**	0.934	**0.933**	0.934	0.933	0.934	0.934
Water consumption	✓	**0.929**	0.929	**0.929**	0.929	**0.929**	**0.929**	0.931	**0.930**	0.931	0.931	**0.931**	0.930

Table S6-2. Estimated impacts for Scenario 1 (forecast of PIC genetics in 2030 compared to 2021) and Scenario 2 (baseline comparison of PIC genetics to the North American industry average, both in 2021) under the PEF-3.1 framework. Impact categories marked with ✓ are among the 14 categories shared by ReCiPe and PEF. Entries in **bold text** are statistically significant ($P < 0.05$)

**Framework PEF-3.1**		PIC 2021 / industry average 2021		PIC 2030 / PIC 2021	
Impact category		cutoff	APOS	cutoff	APOS
Acidification	✓	**0.919**	**0.919**	**0.924**	**0.924**
Carcinogenic toxicity	✓	0.926	0.927	0.931	0.931
Carcinogenic toxicity, inorganics		0.927	0.927	0.933	0.933
Carcinogenic toxicity, organics		**0.925**	**0.925**	**0.927**	0.927
Fine particulate matter	✓	**0.921**	**0.921**	**0.926**	**0.926**
Fossil fuel resources	✓	**0.923**	**0.923**	0.911	0.911
Freshwater ecotoxicity	✓	**0.926**	**0.926**	**0.933**	0.933
Freshwater ecotoxicity, inorganics		0.921	0.921	0.924	0.924
Freshwater ecotoxicity, organics		**0.926**	**0.926**	**0.934**	**0.934**
Freshwater eutrophication	✓	**0.927**	**0.927**	**0.932**	**0.932**
Global warming (GWP100)	✓	**0.925**	**0.925**	**0.930**	**0.930**
Global warming (GWP100), biogenic		**0.927**	**0.927**	0.933	0.933
Global warming (GWP100), fossil		**0.925**	**0.925**	**0.929**	**0.929**
Global warming (GWP100), LULUC		**0.928**	**0.929**	**0.932**	**0.932**
Ionizing radiation	✓	0.937	0.937	0.878	0.878
Land use	✓	0.923	0.923	0.922	0.922
Marine eutrophication	✓	**0.926**	**0.926**	**0.933**	**0.933**
Mineral resources	✓	**0.928**	**0.928**	0.935	**0.935**
Non-carcinogenic toxicity	✓	0.927	0.927	0.934	0.934
Non-carcinogenic toxicity, inorganics		0.927	0.927	0.934	0.934
Non-carcinogenic toxicity, organics		**0.927**	**0.927**	**0.934**	**0.934**
Ozone depletion	✓	**0.923**	**0.923**	0.916	0.916
Photochemical oxidant formation		**0.928**	**0.928**	**0.932**	**0.932**
Terrestrial eutrophication		**0.919**	**0.919**	**0.924**	**0.924**
Water use	✓	**0.918**	**0.919**	**0.907**	0.907

Table S6-3. Estimated impacts for Scenario 1 (forecast of PIC genetics in 2030 compared to 2021) and Scenario 2 (baseline comparison of PIC genetics to the North American industry average, both in 2021) under the IPCC-2021 framework. The impact category marked with ✓ is among the 14 categories shared by ReCiPe and PEF. All entries are statistically significant (P < 0.05)

**Framework IPCC-2021**		PIC 2021 / industry average 2021		PIC 2030 / PIC 2021	
Impact category		cutoff	APOS	cutoff	APOS
AGTP-100		**0.925**	**0.925**	**0.929**	**0.929**
AGTP-50		**0.925**	**0.925**	**0.929**	**0.929**
AGWP-100		**0.925**	**0.925**	**0.929**	**0.929**
AGWP-20		**0.926**	**0.926**	**0.930**	**0.930**
AGWP-500		**0.925**	**0.925**	**0.928**	**0.928**
CGTP-100		**0.927**	**0.927**	**0.932**	**0.932**
CGTP-50		**0.927**	**0.927**	**0.932**	**0.932**
GTP-100		**0.925**	**0.925**	**0.929**	**0.929**
GTP-50		**0.925**	**0.925**	**0.929**	**0.929**
GWP-100	✓	**0.925**	**0.925**	**0.929**	**0.929**
GWP-20		**0.926**	**0.926**	**0.930**	**0.930**
GWP-500		**0.925**	**0.925**	**0.928**	**0.928**

Supplementary S7. Extrapolation of the industry average to 2030.

For the comparison of PIC versus the North American industry average of 2030, a logarithmic regression was fitted to the pre-2021 North American time trends of the relevant KPI traits (i.e. growth rate, feed intake, mortality rate, litter size etc.) as reported by Interpig (2021), MetaFarms (2021), PigCHAMP (2023) and AgriStats, and the fitted patterns were extrapolated to 2030. Figure S7-1 shows these patterns together with the pre-2021 and forecasted PIC trends.

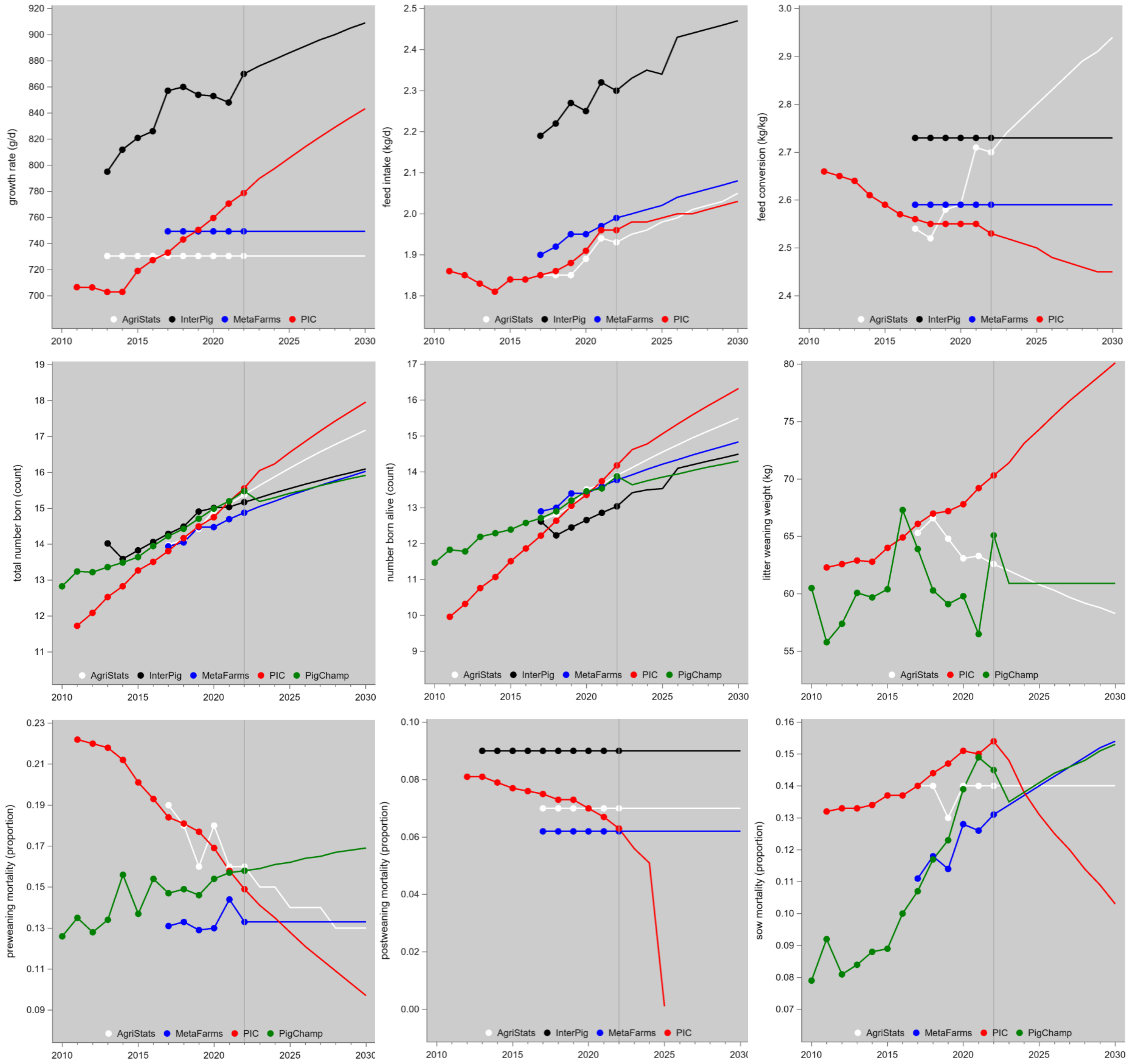

Figure S7-1. The data points up to 2021 show North American time trends of growth rate, feed intake, feed conversion ratio and postweaning mortality of growing-finishing pigs, and of litter size (total number born and number born alive), litter weaning weight, preweaning mortality and sow mortality of reproductive sows, as recorded by four data aggregators (Interpig, MetaFarms, PigChamp and AgriStats), and by PIC on its customer farms. The 2022-2030 trendlines for the data aggregators are extrapolations of the earlier data points, estimated by logarithmic regression of the respective traits on time. The 2022-2030 trendlines for PIC represent the genetic change as predicted by selection index theory from the weighting of each trait in PIC's current (2022) breeding goals, the selection intensity on the associated selection indexes, and the genetic covariances among the traits; see section 2.4. Note that the three mortality traits are categorical (0-1) traits so that their variance depends directly on their incidence p as p × (1 – p); when the incidence is reduced due to selection, the genetic covariances will change and this was not incorporated into the 2022-2030 predictions for PIC genetics.
